# Association of cerebral venous thrombosis with recent COVID-19 vaccination: case-crossover study using ascertainment through neuroimaging in Scotland

**DOI:** 10.1186/s12879-021-06960-5

**Published:** 2021-12-20

**Authors:** Paul M. McKeigue, Raj Burgul, Jen Bishop, Chris Robertson, Jim McMenamin, Maureen O’Leary, David A. McAllister, Helen M. Colhoun

**Affiliations:** 1 Usher Institute, College of Medicine and Veterinary Medicine, University of Edinburgh, Teviot Place, Edinburgh, EH8 9AG Scotland UK; 2 Public Health Scotland, Meridian Court, 5 Cadogan Street, Glasgow, G2 6QE Scotland UK; 3 Institute of Health and Wellbeing, University of Glasgow, 1 Lilybank Gardens, Glasgow, G12 8RZ Scotland UK; 4 Forth Valley Royal Hospital, Larbert, FK5 4WR Scotland UK; 5 Department of Mathematics and Statistics, University of Strathclyde, 16 Richmond Street, Glasgow, G1 1XQ Scotland UK; 6 Institute of Genetics and Cancer, College of Medicine and Veterinary Medicine, University of Edinburgh, Western General Hospital Campus, Crewe Road, Edinburgh, EH4 2XUC Scotland UK

**Keywords:** COVID-19, Vaccination, Cerebral venous thrombosis, Case-crossover studies

## Abstract

**Background:**

To investigate the association of primary acute cerebral venous thrombosis (CVT) with COVID-19 vaccination through complete ascertainment of all diagnosed CVT in the population of Scotland.

**Methods:**

Case-crossover study comparing cases of CVT recently exposed to vaccination (1–14 days after vaccination) with cases less recently exposed. Cases in Scotland from 1 December 2020 were ascertained through neuroimaging studies up to 17 May 2021 and diagnostic coding of hospital discharges up to 28 April 2021, linked to national vaccination records. The main outcome measure was primary acute CVT.

**Results:**

Of 50 primary acute CVT cases, 29 were ascertained only from neuroimaging studies, 2 were ascertained only from hospital discharges, and 19 were ascertained from both sources. Of these 50 cases, 14 had received the Astra-Zeneca ChAdOx1 vaccine and 3 the Pfizer BNT162b2 vaccine. The incidence of CVT per million doses in the first 14 days after vaccination was 2.2 (95% credible interval 0.9 to 4.1) for ChAdOx1 and 1 (95% credible interval 0.1 to 2.9) for BNT162b2. The rate ratio for CVT associated with exposure to ChAdOx1 in the first 14 days compared with exposure 15-84 days after vaccination was 3.2 (95% credible interval 1.1 to 9.5).

**Conclusions:**

These findings support a causal association between CVT and the AstraZeneca vaccine. The absolute risk of post-vaccination CVT in this population-wide study in Scotland was lower than has been reported for populations in Scandinavia and Germany; the explanation for this is not clear.

**Supplementary Information:**

The online version contains supplementary material available at 10.1186/s12879-021-06960-5.

## Background

The first reports in international media of an association of COVID-19 vaccines with thrombotic events appeared on 7 March 2021, when the Austrian Federal Office for Safety in Health Care announced that it had suspended use of a batch of AstraZeneca ChAdOx1 vaccine after cases of thromboembolic events. On 7 April 2021 the UK Medicines and Healthcare products Regulatory Agency (MHRA) and the Joint Committee on Vaccines and Immunisation concluded that there was “a possible link” between the vaccine and cerebral venous thrombotic events and issued new guidance [1]. By 18 May 2021 two countries in the EU/ EEA had discontinued use of the vaccine and 15 had limited its use to older age groups [2]. In the US cerebral venous thrombosis has also been reported following administration of the AD26.COV2.S Johnson & Johnson (JJ) vaccine which like the AstraZeneca ChAdOx1 vaccine utilises recombinant adenoviral vectors encoding the SARS-CoV-2 spike protein [3]. An underlying syndrome denoted “vaccine-induced immune thrombotic thrombocytopenia” (VITT) [4, 5] or “thrombosis with thrombocytopenia syndrome” (TTS) [6] was described, consisting of thrombosis, thrombocytopenia and platelet-activating antibodies to platelet factor 4–polyanion complexes. It was recognized that the spectrum of this syndrome may include thrombocytopenia alone or thrombosis without thrombocytopenia.

Risk/benefit assessments of the use of the AstraZeneca ChAdOx1 vaccine depend on estimating the incidence of CVT and TTS syndrome post-vaccination and evaluating evidence for causality. However, most such estimates have come from voluntary reporting schemes such as the MHRA Yellow Card scheme. These reporting schemes have limitations: vaccine-associated cases may be under-ascertained if the connection with vaccine exposure is not made by the patient or clinician and, once there has been publicity about any possible association with a particular medicine, reporting can be biased thereafter. Although studies based on linking vaccination records to hospital discharge records ascertained either directly [7] or via primary care [8] are less subject to reporting bias, these estimates of incidence depend on the accuracy of coding of hospital diagnoses. Therefore here we took advantage of a nationwide database that captures radiologists’ reports of all imaging studies conducted in the National Health Service (NHS) in Scotland to obtain an unbiased and comprehensive ascertainment of diagnosed CVT since the start of the vaccination programme, linked to the national vaccination database. The aim of this study was to understand the epidemiology of CVT and its association with COVID-19 vaccination. The specific objectives were:

1. to estimate incidence of diagnosed primary acute CVT in Scotland during the vaccination programme
2. to ascertain whether diagnostic coding of hospital discharges or deaths adequately captures diagnosed CVT and allows differentiation of secondary and chronic cases from primary acute cases.
3. to investigate the association of diagnosed CVT with vaccination.

## Methods

In brief, we retrieved all potentially relevant scan reports from the nationwide Picture Archiving and Communication System (PACS) to identify diagnoses of CVT from the study start date of 1 December 2020 up to 17 May 2021. The scan reports contain a summary of the clinical findings, the radiologist’s initial report, and a review by the consultant if the first radiologist was not a consultant. To allow the sensitivity and specificity of discharge coding to be assessed against scan reports as ground truth, we also retrieved all hospital discharge and death records with diagnostic codes for CVT in this time period. As all health care records in Scotland use the Community Health Index (CHI) as a unique identifier, we were able to link PACS reports and hospital discharge and death records to the daily updated national vaccination programme database held by Public Health Scotland/National Services Scotland. We pre-specified a case-crossover design that would compare exposure to vaccine in a recent time window (1 to 14 days since vaccination) with exposure in a less recent time window. The methods are described in detail below.

### Vaccination doses

The COVID-19 vaccination programme in Scotland began on 8 December 2021. Initial priority groups included care home residents and staff, front line health and social care workers and clinically extremely vulnerable individuals. Initially the Pfizer BNT162b2 product (hereafter Pfizer vaccine) was used. From 8 January 2021 the AstraZeneca ChAdOx1 product (hereafter AstraZeneca vaccine) was introduced and from 7 April 2021 the Moderna mRNA-1273 product (hereafter Moderna vaccine) was introduced. CHI number, date of administration, age at vaccination, vaccine product name, whether first or second dose were extracted for all 6894008 vaccination records from 4 December 2020 to 15 July 2021 from the national vaccination database.

### Ascertainment of CVT from CT scan and MRI reports

The key first-line investigation for patients presenting with any acute neurological syndrome is a non-contrast computed tomography (CT) scan of the head. However, this is positive in only about 30% of patients with CVT [9]. For this reason current guidance recommends CT or magnetic resonance (MR) venography in all patients where CVT is suspected [10, 11] unless the initial CT shows strong evidence of CVT and a venogram is contra-indicated.

**Fig. 1:**
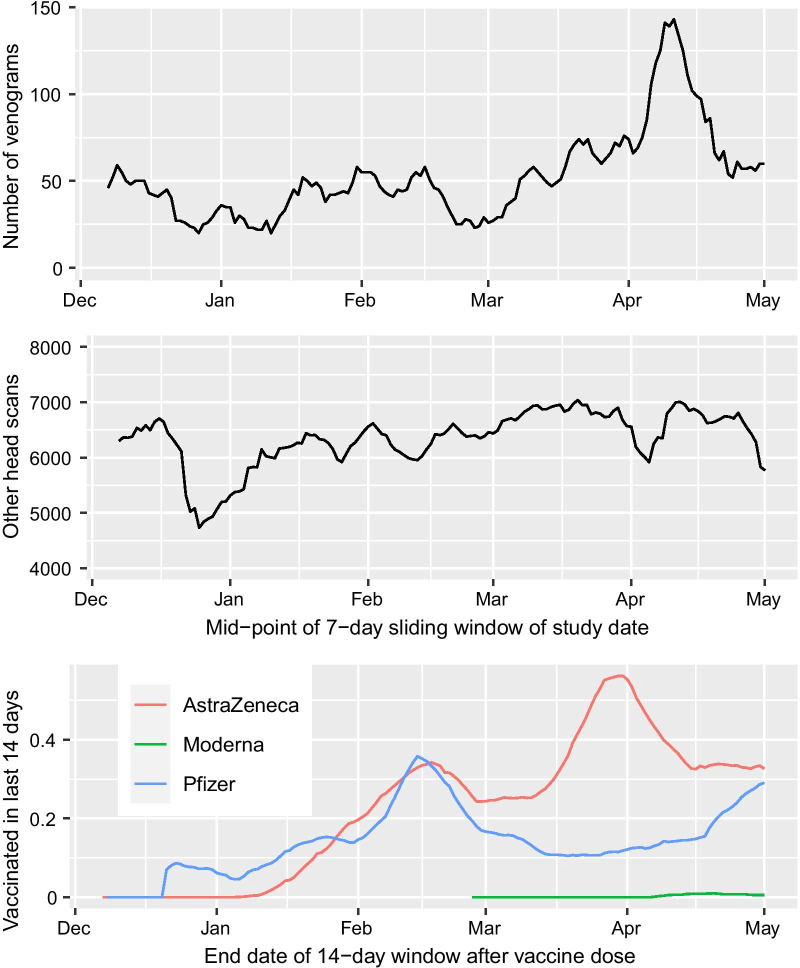
**a** Venograms in the 11 PACS boards by 7-day sliding window of dates; **b** other head scans; **c** population recently exposed: millions vaccinated in last 14 days

Additional file 1: Fig. S1 shows a flow diagram of the process of neuroimaging report extraction and processing. In the pilot stage of the project 13 April 2021 we initially restricted the query of the PACS database to records where the study type (RIS_CODE field) was coded as CT or MR venogram. Comparison with hospital discharge records indicated that this query was insensitive. After discussion with radiologists the query was therefore broadened to extract reports with a wider range of potentially relevant study type codes as listed in Additional file 1 and any reports with a study modality of CT or MRI where the study description field contained any of the strings “head”, “brain” or “cerebr”. All such reports of CT and MRI studies from 1 Dec 2020 to 17 May 2021 were retrieved from the PACS database.

For three of the 14 health boards in Scotland, the PACS database contained the radiology report only as an image that could not be queried. For these boards extractions were carried out directly from the local radiology information system that feeds the national PACS instead. For Lanarkshire (12.1% of the Scottish population) the same query was run as for the national PACS . From the two other boards that do not encode reports as text in PACS—Forth Valley and Dumfries and Galloway, covering 8.3% of the Scottish population—only the initial RIS code query for venograms up to 13 April 2021 was completed; a subsequent broader query could not be obtained at the time of this study.

The extracted scan reports were filtered to retain any scan likely to be informative for the presence of CVT i.e. all those with a study type or study description including a venogram or where any of the following strings appeared in the the radiologist’s report: “sinus thrombosis”, “sinus thrombus”, “venous thrombosis”, “venous thrombus”, “venogr”. For any individual with at least one scan meeting these criteria all scan reports for that person since 1 Dec 2020 were retained for review.

After arraying scans for each individual chronologically, each clinical event was scored by a doctor as primary acute, possible, follow-up, chronic, no valid result, secondary or negative (see Additional file 1). A new clinical event was defined by a new illness leading to a hospital visit. The date of onset of the event was assigned as the date of onset of symptoms where this was recorded in the scan report, otherwise as the earliest date of hospital admission or scan. The reviewing doctor (HC) had no access to vaccination status except where a mention of this was embedded in the scan report. A second doctor (DM with adjudication by RB) re-scored all scans that had been coded non-negative and a random sample of 30 negative scans, with the scoring of the first reviewer masked.

### Ascertainment of CVT from discharge diagnoses and death certificates

Records of all hospital discharges in Scottish Morbidity Record 01 (SMR01) from 1 December 2020 to 2021-04-28 and National Register of Scotland death registrations from 1 December 2020 to 2021-04-16 were queried for any mention of a CVT code as discharge diagnosis or cause of death. The ICD-10 codes used were: I63.6 (cerebral infarction due to cerebral venous thrombosis, nonpyogenic); I67.6 (nonpyogenic thrombosis of intracranial venous system); and G08 (intracranial and intraspinal phlebitis and thrombophlebitis). Records with mention of a local secondary cause such as infection, intracranial abscess or brain tumour were excluded by matching of the regular expression

${{^{\hat{\,}}}}$A|${{^{\hat{\,}}}}$B|${{^{\hat{\,}}}}$C7[012]|${{^{\hat{\,}}}}$D3[23]|G06[02] in any of the diagnostic codes.

### Statistical methods

The case-crossover design compares event rates in time windows of recent and less recent exposure; this is equivalent to a matched case-control study in which the case and the control are the same person in different time windows [12]. We pre-specified the time window for recent exposure to vaccine as 1 to 14 days before the onset of symptoms as given in the summary of the clinical history in the scan report.

To allow comparison with other studies that have used a 28-day time window, we also tabulated events and rates for the period 15–28 days. All post-vaccination cases were within 84 days of vaccination, so for the case-crossover analysis the 1–14 day time window is compared with the 15–84 day time window. For each vaccine product and each time window, the number of person-fortnights at risk was calculated to the end of follow up. To allow for the variation between health boards in latest date of case ascertainment via scans, the person-days in each exposure category from 14 April to 17 May 2021 were multiplied by 0.917 (the proportion of the population not covered by Forth Valley or Dumfries and Galloway where only records up to 14 April were extracted) and the person-days from 17 May 2021 to 1 June 2021 were multiplied by 0.121 (the proportion covered by Lanarkshire where records up to 1 June 2021 were extracted).

As the numbers of observed events were too small for confidence intervals to be equivalent to credible intervals, Bayesian credible intervals were calculated directly from the quantiles of the posterior distribution. With a flat prior on the logarithm of the event rate and $r$ observed events in $n$ million person-fortnights, the posterior distribution of the event rate is a gamma distribution with shape parameter $r$ and inverse scale parameter $n$. Conditional on the total number of events, the likelihood given $r_1$ events in $n_1$ person-days in the recently exposed time window and $r_0$ events in $n_0$ person-days in the less recently exposed time window is $p^{r_1}(1 - p)^{r_0}$ where $p = n_1 e^\beta /(n_0 + n_1 e^\beta )$ and $\beta$ is the log rate ratio. With a flat prior on the log rate ratio $\beta$, normalizing this likelihood as a function of $\beta$ gives the posterior distribution of $\beta$.

## Results

### CVT case ascertainment

As shown in Additional file 1: Fig. S1, 142,964 neuroimaging reports pertaining to 80905 individuals were retrieved. After de-duplication and retention of all venogram reports, and any reports containing keywords as described in the Methods section, 2760 scans pertaining to 1607 individuals were retained for manual coding. Table 1 shows the codes assigned to these individuals after review. The independent coding validation by a second masked coder resulted in only one reassignment where a case with metastatic cancer but no brain metastases was reassigned as primary acute rather than secondary. 48 cases ascertained through scans were coded as having a primary acute CVT after 1 December 2020, and 18 were coded as “possible CVT”. No additional cases in the study period were ascertained through death certificates with CVT codes.

**Table 1:** Scoring of CVST diagnostic categories on 1605 individuals with scans after 1 December 2020 that were filtered by study description or text strings in report for manual review

	Number of individuals
Primary acute	48
Possible	18
Chronic	19
Follow-up	10
No valid result	0
Secondary	33
Negative	1479
All	1607

The yield of cases through SMR01 discharge reports up to the latest discharge in the SMR01 extract is shown in Table 2 In the extract used for this study there were 34 individuals with first mention after 1 December 2020 of a CVT code, of whom 29 had no mention of a secondary code on any discharge record. Of these 29, 7 had neuroimaging reports that showed a secondary cause, chronic thrombus, or stenosis rather than thrombus. Only 3 of the 29 events ascertained from SMR01 discharge records had no neuroimaging report retrieved by the PACS query. Of these one had an unrelated condition as main diagnosis, one had a CVT code as main diagnosis but had stayed only one night in hospital, and one had a CVT code as main diagnosis and a scan record but no report, from one of the two health boards that did not include the text of scan reports in the PACS database. On the basis that CVT diagnostic code as main condition had high specificity (14/17) the two cases with CVT coded as main condition were retained as primary acute. As none of these cases without scan reports were post-vaccination, their inclusion or exclusion does not affect the case-crossover results.

**Table 2:** 29 SMR01 discharges with CVT diagnoses from 1 December 2020 by scan report

	Primary acute	Chronic/secondary/negative	No scan	All
As main condition	14	3	2	19
As other condition	5	4	1	10
Total	19	7	3	29

Ascertainment based on mention of CVT codes G08, I63.6, I67.6 excluding

records with mention of codes for secondary causes as described in

Methods

Table 3 shows that of the 48 primary acute CVT cases ascertained through neuroimaging, 19 had a discharge record with a CVT code, 8 had a discharge record with no CVT code, and 21 had no discharge record. Note that as coding of hospital admissions is done after discharge with varying lags, no exact cutoff date for the latest date of admission of cases ascertained through SMR01 can be defined. However as shown in 3 of 35 primary acute CVT case ascertained through PACS up to the end of the first quarter of 2021 (a period probably fully captured by the SMR01 extraction), only 17 had had a CVT diagnosis code on an SMR01 record.

**Table 3:** 48 scans coded as primary acute by 3-month calendar period and mention of a CVT code on discharge record

	CVT diagnosis	Other diagnosis	No coded discharge record	All
2020-Q4	8	1	2	11
2021-Q1	9	7	8	24
2021-Q2	2	0	11	13
Total	19	8	21	48

Combining the 49 cases ascertained through scans and scored as primary acute with the two additional cases with CVT coded as main diagnosis that had been ascertained only through SMR01, 50 cases classified as primary acute were retained for analysis, together with 18 scan-ascertained cases coded as possible.

### Relation of CVT to vaccine exposure

Table 4 shows the number of doses by age group and vaccine product from 1 Dec 2020 to 2021-07-15. The vaccine product was missing for about 0.4% of recorded doses.

**Table 4:** Vaccine doses by age group up to 2021-07-15

	Pfizer	AstraZeneca	Moderna	Total doses
0–39	1,035,856 (39%)	378,507 (9%)	144,157 (84%)	1,558,520 (23%)
40–59	690,208 (26%)	1,837,077 (46%)	27,208 (16%)	2,554,493 (37%)
60 or more	963,420 (36%)	1815596 (45%)	862 (1%)	2,779,878 (40%)
–	4 (0%)	1113 (0%)	0 (0%)	1117 (0%)
Total doses	2,689,488	4,032,293	172,227	6,894,008

Table 5 tabulates the post-vaccination cases by vaccine product and time since vaccination. As pre-specified, the time window of 1 to 14 days was used to define recent exposure. All other events in vaccinated individuals occurred between 15 and 84 days after vaccination. Case-crossover estimates of rate ratio were based on comparing the 1–14 day time window with the 15–84 day time window. In each time window, the number of person-fortnights exposed in that time window is the denominator from which the rate is calculated.

**Table 5:** CVT cases after vaccination, by time since vaccine dose

Product	Days since vaccination			Rate ratio
	1–14	15–28	29–84	1–14 vs 15–84
Person-fortnights exposed				
Pfizer	1,908,751	1,784,505	4,942,003	–
AstraZeneca	3,154,182	2,698,048	7,390,991	–
Moderna	23,578	17,801	21,013	–
Cases scored as primary acute				
Pfizer	2	1	0	–
AstraZeneca	7	2	5	–
Moderna	0	0	0	–
Rate (95% credible interval) primary acute per million person-fortnights				
Pfizer	1 (0.1 to 2.9)	0.6	0	7 (0.6 to 95.8)
AstraZeneca	2.2 (0.9 to 4.1)	0.7	0.7	3.2 (1.1 to 9.5)
Moderna	0	0	0	–
Cases scored as primary acute or possible				
Pfizer	2	3	1	–
AstraZeneca	9	3	7	–
Moderna	0	0	0	–
Rate (95% credible interval) primary acute or possible per million person-fortnights				
Pfizer	1 (0.1 to 2.9)	1.7	0.2	1.8 (0.2 to 8.9)
AstraZeneca	2.9 (1.3 to 5)	1.1	0.9	2.9 (1.1 to 7.2)
Moderna	0	0	0	–

Seventeen definite CVT events and 25 definite or possible CVT events occurred post vaccination: the others were in individuals who had not been vaccinated at the time of onset. Of the 7 cases with onset within 14 days of exposure to AstraZeneca vaccine, 6 were in people aged less than 60 years and 4 were in women.

The observed rate of definite (primary acute) CVT diagnoses in the 14 days after exposure to AstraZeneca vaccine is estimated as 2.2 (95% credible interval 0.9 to 4.1) per million doses. For the Pfizer vaccine, the corresponding rate was 1 (95% credible interval 0.1 to 2.9) per million doses. There were no cases in the small numbers exposed to Moderna vaccine. For comparison, the observed rate of primary acute CVT in the 15–84 day time window after exposure was 12 per million per year (95% credible interval (5.3 to 22.2). The rate ratio associated with exposure in the last 14 days to the AstraZeneca vaccine was estimated as 3.2 (95% credible interval 1.1 to 9.5) for primary acute CVT, and 2.9 (95% credible interval 1.1 to 7.2) for primary acute or possible cases.

For comparison with other studies that used a 28-day time window, the rate of primary acute CVT within 28 days of vaccination with the AstraZeneca product, based on 9 events in 2926114 28-day time intervals, was 3.1 (95% credible interval (1.4 to 5.4) per million doses. In those aged under 60 years the rate, based on 8 events in 1319795 28-day time intervals was 7 (95% credible interval (3.1 to 11.9) per million doses.

### Checking for bias in case ascertainment

Figure 1 shows the total number of venograms (panel a) and other head scans (panel b) in boards with complete ascertainment using PACS, by 7-day sliding window of study date. The average number of venogram studies was about 50/day from December 2020 to March 2021, but increased sharply in the first two weeks of April to a peak of nearly 150/day day in mid-April, around the time that the association of CVT with vaccination first received wide publicity in the UK. However the number of individuals exposed to the AstraZeneca vaccine in the last 14 days had peaked earlier in late March. Of the 9 cases that occurred within 28 days of vaccination with the AstraZeneca product, 6 were before April 2021, when the increase in number of venograms per day began.

## Discussion

### Statement of principal findings

In this population-wide study we have captured all diagnosed CVTs nationally since before the start of the vaccination programme. We have shown that ascertainment of CVTs from diagnostic codes on hospital discharge records has low sensitivity for CVT; systematic review of scan reports is required for complete ascertainment of diagnosed cases and to distinguish primary acute from secondary cases. The number of events in those recently exposed to vaccine is small but we can still calculate a credible interval for the incidence rate and thus an upper bound. For the first 14 days after AZ vaccination, we calculate an upper bound of 4.9 per million doses on the absolute rate, and 9.1 for the rate ratio. Although the rate of venogram studies increased markedly in early April 2021 after the association of CVT with vaccination had received wide publicity, most of the vaccine-associated cases in this study were diagnosed before this, and thus it is unlikely that ascertainment could have biased the association with recent vaccine exposure. When combined with prior evidence of excess risk of CVT associated with the AstraZeneca vaccine in other populations, the case-crossover analysis supports a causal interpretation of this association. Although the number of cases exposed to the Pfizer vaccine is too small for any comparison between the two products, an upper bound can still be placed on the absolute risk associated with the Pfizer vaccine.

### Strengths and limitations of this study

A strength of this study is the complete ascertainment of cases in the population, not reliant on adverse event reporting or diagnostic coding of hospital discharge records. Manual review of scan reports, which typically included a summary of the clinical history allowed some CVT cases to be recoded as secondary to a local lesion such as a tumour or as chronic rather than acute CVT, and allowed the date of onset to be determined from the summary of the clinical history. Accurate assignment of date of onset is required for the case-crossover analysis to be valid. Frequently the onset of symptoms was a few days before presentation to hospital. The time window for onset of vaccine-associated CVT may thus be overestimated in studies that have reported the median time from vaccination to hospitalisation to be more than 10 days [13, 14].

The case-crossover design eliminates confounding by time-invariant factors, which may be strong where vaccine allocation is based on pre-existing risk conditions that were used to allocate priority for vaccination, and thus provides evidence for causality where an association is detected. Note that our analysis assumes an ignorance prior; it ignores any prior evidence of association. Unlike the self-controlled case series design, which has also been used to study the associations of adverse events with COVID-19 vaccine [15], the case-crossover design can be used to study events such as CVT that are likely to affect the probability of being vaccinated within the next few weeks.

Limitations of our study are that we do not have access to data on platelet counts, D-dimer or platelet factor 4 antibody levels, allowing enumeration of the number of CVT events that are part of the formally defined VITT/TTS syndrome as meeting associated haematological criteria. We note that the Brighton Collaborative however acknowledges that the TTS syndrome definition may be too restrictive by excluding isolated thrombotic events that are causally related to vaccine. Also we did not ascertain venous thromboses at sites other than brain.

### Relation to other studies

From this study the background incidence of primary acute CVT in Scotland (excluding the 14-day time window after vaccination) can be estimated as about 12 per million adults per year: this is similar to the estimate of 16 per million per year in Australia in 2016 [16] based on ascertainment of cases via neuroimaging records. Most other estimates of CVT incidence have relied on ascertainment via diagnostic coding in health informatics systems, which is likely to underestimate the incidence of CVT. A recent study from Scotland based on primary care, hospitalisation and death records in the total population reported 19 CVT events between 8 December 2020 and 14 April 2021 [8]. For the same period our study ascertained 41 primary acute cases of CVT in Scotland.

Adverse event reporting systems can give early warning of unexpected effects but cannot be relied on to estimate incidence rates. By early April 169 CVTs had been reported to the EMA by which time about 34 million doses of Astra Zeneca vaccine had been administered, giving a rate of about 5 per million doses. In the UK as of 9 June 2021 the Yellow Card scheme operated by the MHRA had received 390 reports of cases of “major thromboembolic events with concurrent thrombocytopenia”. Cerebral venous sinus thrombosis was reported in 140 of these. By 2 June 2021 30.1 million doses had been administered, so allowing for a 1-week lag the incidence was 13.0 per million doses for the syndrome and 4.7 per million doses for CVT [17]. A subset of 102 of these cases were reported in more detail by haematologists from 96 hospital trusts detailed study: the denominator was estimated as 24 million doses, giving a rate of 4.3 per million doses in the first 30 days [14]. Our estimate of 3.5 per million doses in the first 28 days is similar to these estimates, indicating that the Yellow Card system, for all its limitations, has not seriously underestimated the incidence of vaccine-associated CVT.

The upper bound of 6.2 per million doses for incidence of CVT within 28 days of vaccination with the AstraZeneca product estimated in this study is rather lower than estimates reported from Scandinavia and Germany. In a study of all 281264 individuals aged 18-65 years in Denmark and Norway who received a first dose of the AstraZeneca vaccine, there were were 7 observed CVT events within 28 days of vaccination compared with 0.3 expected from rates in the general population [7]: a rate of 25 per million doses. A study of CVT events ascertained through neurologists from nine states in Germany where 2320535 first doses had been administered reported 27 cases within 31 days of AstraZeneca vaccine recipients, giving a rate of 15 per million doses within 31 days of vaccination [18]. In those aged under 60 years the rate in the German study was 18 per million doses, compared with 7 per million doses for the same age group and 28-day time interval. The reasons for this difference between the UK and EU countries are not clear. Process-related impurities have been reported in batches of the AstraZeneca vaccine [19]. A report from the European Medicines Agency dated 24 March 2021 stated that they had requested AstraZeneca to provide “a full batch analysis for specific lots and batch data from UK supplied lots to understand if there are any clear differences between that and the EU products” [20].

## Conclusions

This study based on complete ascertainment of CVT cases makes it possible to set a definitive upper bound on the rate of vaccine-associated CVT in Scotland. The results reinforce the importance of establishing comprehensive surveillance of adverse events occurring after vaccination. By using e-health record systems we were able to obtain all neuroimaging reports for the population and to report preliminary results to public health agencies within a few weeks of initiating this study. This entailed labour-intensive manual coding of scan reports. For longer-term surveillance and scaling to larger populations a natural language processing algorithm could be developed to identify CVTs in imaging reports, but some manual coding would still be required especially to assign the date of onset.

Policy on the continued use of the AstraZeneca vaccine has been driven by estimates of the risk/benefit ratio, with risk of TTS estimated from adverse event reporting schemes [21]. Thus on 7 May 2021 the Joint Committee on Vaccination and Immunisation advised that those under age 40 should be offered an alternative to the AstraZeneca vaccine [22]. Evaluating the risk / benefit ratio of COVID-19 vaccination in healthy young adults and children depends on being able to detect rare adverse events post-vaccination through surveillance, so that the risk of such events can be compared with the low risk of severe complications of COVID-19 in these groups.

## Supplementary Information

**Additional file 1: Figure S1.** Flow diagram for case ascertainment from scan reports.

## Data Availability

The component datasets used here are available via the Public Benefits Privacy Panel for Health at https://www.informationgovernance.scot.nhs.uk/pbpphsc/ for researchers who meet the criteria for access to confidential data. All source code used for derivation of variables, statistical analysis and generation of this manuscript is available on https://github.com/pmckeigue/covid-scotland_public.

## Abbreviations

CVT: cerebral venous thrombosis
VITT: vaccine-induced immune thrombotic thrombocytopenia
TTS: thrombosis with thrombocytopenia syndrome
PACS: picture archiving and communications system
MHRA: Medicines and Healthcare products Regulatory Agency
