# Supplementary material for: Association of cerebral venous thrombosis with recent COVID-19 vaccination: case-crossover study using ascertainment through neuroimaging in Scotland
Source: BMC Infect Dis. 2021 Dec 20;21:1275. doi: 10.1186/s12879-021-06960-5 (PMC8686098; doi:10.1186/s12879-021-06960-5)
Supplement: Supplementary file 1 — Additional file 1: Figure S1. Flow diagram for case ascertainment from scan reports. [file 12879_2021_6960_MOESM1_ESM.pdf]

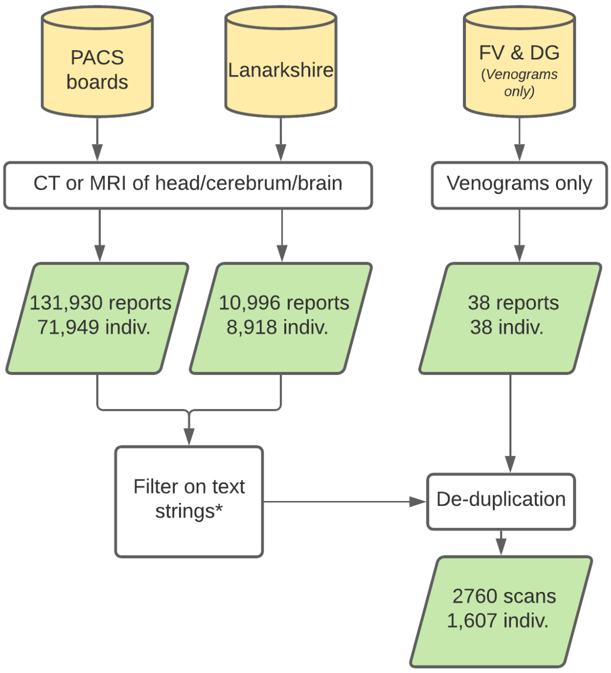

* Retained all venograms as well as any reports containing any of the following text:- sinus thrombosis, sinus thrombus, venous thrombosis and/or venous thrombus.

Fig S1. Flow diagram for case ascertainment from scan reports

PACS query for ascertainment of scans possibly informative for CVT

- (Modality=CT OR Modality=MR) AND (Study description contains “head” or ”cerebr*” or “brain”)

OR

- RIS code = ANY OF
  - CVENO CT Venogram
  - CVEIC CT Venogram Intracranial
  - CVECE CT Venogram cerebral
  - CTHVG CT Head Venogram
  - MVENO MRV Venogram
  - CBNTA CT Brain neck thorax Abdo and pelvis
  - CHAP CT Head abdomen and pelvis
  - CHAPC CT Head abdomen pelvis with contrast
  - CHNTAP CT Head neck thorax abdomen and pelvis
  - CHTA CT Head thorax and abdomen
  - CHTAP CT Head thorax abdomen and pelvis
  - CHTAPC CT Head thorax Abdo pelvis with contrast
  - CHTH CT Head and thorax
  - CHTHAC CT Head thorax abdomen with contrast
  - CHTHC CT Head and thorax with contast
  - CSKNE CT Head and neck
  - CSKPE CT Brain perfusion study
  - CSKUH CT Head
  - CSKUHC CT Head with contrast
  - CSKUC CT Head with contrast
  - MAICA MRA Head
  - MAICAC MRA Head with contrast
  - MBRCC MRI Brain and cervical cord
  - MSKPE MRI Head brain perfusion study
  - MSKUH MRI Head
  - MSKUHC MRI Head with contrast
  - MSKUS MRI Head spectroscopy
  - MVSKU MRV Cerebral veins

Protocol for scoring neuroimaging studies

Scoring at scan level

- **negative** - no evidence of any venous sinus thrombosis in the report
- **secondary** - evidence of acute or chronic CVT but there is another local pathology contributing such as meningioma, mastoiditis, sinusitis or encephalitis. Systemic factors such as Factor V Leiden deficiency, post-partum state, use of oral contraceptives, or pro-thrombotic drugs are not classified as secondary causes. Trauma caused by a external event such as traffic accident or assault is classified as a

secondary cause, but history of a fall that could have resulted from a primary brain event is not classified as a secondary cause.

- **chronic** - this code is assigned when there is a new presentation but the scan shows evidence of unchanged or resolving thrombosis or recanalising thrombosis. There may or may not be a definitive history of an earlier acute primary event.
- **follow-up** - this code is assigned where the scan shows evidence of thrombosis but has been done as a follow up of a prior event either during the same hospitalisation or later as a routine follow-up. This code was assigned to reports without the primary scan necessarily being available at time of report coding.
- **possible** - this code is used where there is some evidence that may suggest thrombosis but which is not definitive; many such reports will recommend further investigation
- **primary acute** - scan reported as consistent with venous thrombosis, not assigned as chronic and which cannot definitely be assigned to other local cause.
- **no valid result** - there is a report but the report text is missing or the scan was declared to be a technical failure or unreadable

Coding at person level

For this process all available scans extracted for an individual were arrayed at person level. An event encompasses all scans pertaining to a new presentation. A follow up scan done several months or years later is a new event.

- **negative** - none of the scans have found evidence of a CVT or an initial scan with possible CVT is followed by a definitive scan such as venogram that rules out CVT
- **secondary** - an event where there is evidence of acute or chronic CVT but there is another local brain pathology contributing such as meningioma or mastoiditis or sinusitis or encephalitis
- **chronic** - there is a new presentation and one of the scans shows chronic thrombus
- **follow-up** - Most events that include follow up scans during that admission will be coded as primary acute events with the date of the event being that of the presentation date of the originating thrombus event. For follow up scans in a separate hospital attendance these are coded as follow up but the event onset date is given as the original primary event date where it is available. Where the primary event is not referred to or given a date the final event code maybe left as follow-up with the date of onset left blank
- **possible** - one of the scans for the event found evidence of a possible CVT and no subsequent scan ruled this out or resolved whether or not a thrombus was present
- **primary acute** – a new presentation where any scan shows changes consistent with thrombus that is not assigned as chronic and is not attributed to a secondary local cause

Examples of how the protocol is applied

1. Patient presents with a seizure – all scans are negative. Two weeks later they present with new onset confusion – CT scan negative, MR venogram shows bleed and CVT . The first admission is coded as negative for each scan and for the event. The second set of scans is coded as negative and primary acute respectively and the event is coded as primary acute.

2. Patient presents with confusion and headache after a fall – CT head has no abnormality , subsequent CT venogram shows filling defect in venous sinus – a scan three days later shows similar picture. The reports are coded as negative, primary acute, follow up. The event is coded as primary acute.
